# Brief Remarks on the Diversities of the Human Species, and on Some Kindred Subjects

**Published:** 1842-12-24

**Authors:** 


*Brief Remarks on the Diversities of the Human Species, and on some Kindred Subjects.* Being an Introductory Lecture delivered before the Class of the Pennsylvania Medical College, in Philadelphia, November 1, 1842. By Samuel George Morton, M.D. Professor of Anatomy and Physiology in that Institution. Published by the Class. 8vo. pp. 24.

This is an admirable essay, showing extensive research, and deep and just thought. We commend it to the profession and to the scientific reader. Professor Morton's ability to treat the present interesting subject will be generally admitted, and expectation will not be disappointed. Reflection has convinced him that a primeval difference has existed among men; "not an accidental occurrence, but a part of that all pervading design, which has adapted man in common with animals and plants, to those diverse conditions which form a necessary part of the economy of creation." These diversities are seen in the physical and intellectual man, and are conspicuous even in his moral character. Is it not probable that the Infinite Power which conducted safely a single favoured family through the dangers of the deluge, should, before their subsequent dispersion in different climes, adapt them "to those varied physical circumstances with which they were henceforward to contend?"

"In the first place, then, we may remark, that if the black complexion was the mere consequence of the action of the sun's rays in a hot climate, the Indians of our own continent, who inhabit the torrid zone, ought to be as dark-skinned as the inter-tropical Africans; which is very far from being the fact, for these American tribes are no darker than others who live on the shores of the Rio de la Plata, in the cold region of Patagonia. Again, if climate caused the peculiar texture of the hair in the African, a similar temperature in the same latitudes of America, ought to produce an analogous result in at least some portion of the indigenous population; but the hair of the Indian, in all its localities, is long and lank, like that of the Mongolian: nor, on the other hand, does there appear to be the smallest tendency in any American climate to change the hair of the Negro; for we have the experience of three centuries in the West India Islands, in disproof of any such mutation.

"If the African derives his complexion from the causes to which we have adverted, how does it happen that he becomes no fairer in a colder climate? Real Negroes constitute the indigenous population of Van Diemen's Land, which is nearly as cold as Ireland; and yet these very people are among the most strongly marked tribes of the African race. In many islands of the Indian Archipelago, the Nigritos, a race of Negroes of small stature, inhabit the hill-country, and the Malays the low-lands nearer the coast; and yet the Malay, under these circumstances, does not approximate to the Negro in any one of his physical characteristics.

"So also the Dutch inhabitants of the Cape of Good Hope, who have lived for three centuries among the Hottentots, have preserved their natural traits unaltered; and this remark is also true of the Arabs who have lived during many generations among the Negroes in the heart of Africa, yet have lost none of the distinctive features of their race. Analogous examples might be multiplied to a great extent; but these may suffice the purpose of present illnstration.

"That climate has a certain and obvious effect on the human body, I most certainly admit; and, taking the skin for an illustration, we all know the effect of the sun's rays in producing a darker complexion, as seen in our own latitudes, and which is more strikingly manifest in those Caucasian or white nations who inhabit inter-tropical regions. They become of a dark brown complexion, not unfrequently as sable as mulattoes; but the children of such parents, if protected from the sun's rays, preserve the complexion which is characteristic of their progenitors, as in the instance of the Arabs to whom

I have already adverted. But, with regard to the permanence of those organic characters which mark the different races of men, a conclusive source of evidence has within a few years been disclosed to us in the monuments of Egypt. These venerable and truly wonderful remains of human ingenuity, embrace numberless legends, of which the knowledge of hieroglyphic literature has established not only the meaning, but the dates themselves; and by their evidence we discover the interesting fact, that the several races of men were as perfectly distinct in Egypt upwards of three thousand five hundred years ago as they are now. The white man and the Negro are there depicted, side by side, with the same physical characteristics which are now familiar to our daily observation. This vast period of thirty-five centuries, has made no appreciable change in either; and as less than seven hundred years could have intervened between the positive existence of these distinctions and the epoch of the Deluge according to the Hebrew chronology, we come to the unavoidable conclusion, as already stated, that these diversities of organization were coeval with the dispersion of our species. Let me repeat, therefore, my admonition, that we may not hastily attribute to mere chance those singular phenomena which bear the impress of obvious and original design.

" Some minds would be perhaps equally disposed to attribute the diversity of languages also to accident, were it not for the positive evidence to the contrary, which is preserved in the inspired records; proving that in this, as in every other instance, whatever was requisite for the protection, variation, and perpetuity of the human race, required but the fiat of Omnipotence, and it was done.

" The negro presents us, moreover, with a remarkable example of the adaptation of constitution to climate. Thus, there are vast tracts on both the eastern and western coasts of inter-tropical Africa, in which the European constitution at once becomes the victim of enervating and destroying fevers: and although it may, for a time, resist the fatal effect of these subtle influences, it sooner or later becomes a prey to them. No precautions can prevent them; and no manner of life accustoms an exotic constitution to contend with its unseen but indomitable enemy. In these very climates, in the midst of these lethal exhalations, in this foul and poisoned atmosphere, the negro reaches the acme of his physical nature, and scorns those precautionary restraints which are necessary to the European.

" This remark applies to the negro not only in his native African regions, to which we presume his constitution is essentially assimilated, but also in exotic climates; for I believe the fact has been satisfactorily proved, that he is much less subject than the white man to the yellow fever of our own country, and also to those destroying epidemics which infest the rice plantations and other marshy districts of the southern states.

" Yet, on the other hand, the native inhabitants of a district of country are liable to diseases from which strangers are exempt, owing to a seemingly primitive difference of constitution which appears to belong to peculiar developments of the physical man. For example, in or about the year 1764, an inflammatory fever broke out among the Indian population of Nantucket and Martha's Vineyard, and was wholly confined to individuals of that race. The whole number of Indians in Nantucket was 340; of these, 258 took the distemper, and only thirty-six recovered. In Martha's Vineyard, it attacked every individual of those Indian families in which it made its appearance, being fatal in nearly all cases. Now mark another striking fact: not a single white person took the disease; and all those individuals who were of a mixed Indian and European origin, although they took the disease, recovered to a man!*

"Do not these and similar facts point to an original adaptation of the physical man to the local position he was ordained to inhabit? I apprehend that without such adaptation, the patriarchal germs of our species would have been utterly destroyed in the effort to contend with those pestilential influences which appear to be inherent to certain localities on the surface of our earth."

Man as an inhabitant of every climate must be capable of existing under every variety of temperature, from parching equational sun, to 70° north latitude, where even mercury freezes. He too must be omnivorous.

"If animal diet was essential to the human economy, China and India, now the most thickly peopled portions of the globe, would of necessity be very sparsely inhabited. These nations, as is well known, live almost exclusively on rice; and yet they enjoy remarkably uniform health, are capable of much fatigue, and live to a good age. Some savage tribes of our own country live for months of the year on roots alone; yet these unfortunate people have all the physical attributes of the cognate tribes. Nay, more: the philosophic traveller, Von Humboldt, met with a people called Ottomacs, living on the shores of the Orinoco, who, he assures us, are compelled by the scantiness of food, to live for several weeks and even months of the year, chiefly on an unctuous clay which contains some nutritive properties, and serves the purposes of life. This seems for a moment, beyond our credence; yet upon how little aliment the human body can be nourished and sustained, even amid the greatest privations, has been often brought to the test of painful experiment in some of those polar expeditions, which have formed a remarkable feature in the history of the present century. Men have been compelled, in the extremity of famine, to eat their very shoes, and have found in them sufficient sustenance to sustain life, and toil and fatigue, for many days together.

"If we next regard the most northern tribes of both continents, we find them living exclusively on animal food, and this in a most gluttonous quantity, and in the most disgusting condition. The Eskimaux and the Kamschatkans, the Ostiaks, Samöides and other polar nations, eat their food raw or half cooked, with equal indifference. And of what does this food consist? The flesh of seals, the blubber of whales, and the visceral parts of various marine animals, and often in a state of absolute decomposition. Yet this diet is not only suited to their palates but adapted to their digestion; for on it they thrive well, grow fat, enjoy life, and are capable of undergoing a degree of fatigue and privation which to us would be appalling indeed.

"Further to the south, yet still in a comparatively inhospitable latitude,

*See Lawrence's Lectures on Zoology, &c. I commend this work, which has been republished in Salem, Massachusetts, to your attentive perusal, as replete with instructive facts, to which I have recurred on the present and all other occasions when engaged in inquiries into the Natural History of Man. The more elaborate work of Dr. Prichard, of Bristol, England, will amply reward an attentive perusal, inasmuch as it embodies the most extended series of facts in Anthropology to be found in our language. This invaluable work has not been reprinted in this country, but can be obtained from our larger libraries.

the Norwegians are seen to grind the bark of certain trees for the purpose of mixing it with their scanty allowances of oat-meal or barley; and yet these very people are scarcely surpassed in health, strength, or longevity, by any other inhabitants of Europe.

"In further proof of the omnivorous nature of man, we have only to regard the civilised communities of which we ourselves form a part. Look at the multiplied varieties of animal and vegetable food which are every day exhibited in our markets, and displayed upon our tables. All nature is taxed to provide this infinite diversity of aliments. The earth, the sea, the air, each is ransacked to contribute something to this grand and necessitous requisition of our nature. Then look again at the multiform culinary operations to which these various articles of diet are subjected, and we are compelled to admit that man is omnivorous in the broadest sense of the word. The truth is, he must be fed. He rises in the morning, whether from his hard and unyielding bed, or from his downy couch, and his first impulse is that of hunger. Whether he works or lives in idleness; whether he fights or philosophises; whether he tills the earth, or writes romances, he must, he MUST be fed.

> Hunger! Thou art in truth a god, to whom
> Men render homage. Daily dost thou drive
> Thy famished legions forth, to toil, or beg,
> Or rob. Who dares contend with thee? Not one.
> Life is a strife for bread.

"And when we reflect that eight hundred millions of human beings feel this daily necessity for food, a necessity which cannot be postponed nor compromised by any crafty subterfuges, how stupendous does that provision appear, which is adequate to the wants of so vast a family!

"But although man, as we have observed; is capable of living on any or all kinds of food; although his aliments are, in some regions, necessarily limited to a very few articles, and these exclusively of the vegetable or of the animal kingdom, yet his mind and body are most perfectly developed when his diet is of a duly mixed character, and adapted to those ever-varying conditions of repose and activity to which he is by nature subject.

"The lower animals have their prescribed localities, their native haunts, their own essential range of climate. What would be the condition of the polar bear if placed under the equator, or the lion if removed to the perpetual snows of the arctic circle? Each would become paralyzed and powerless, and sink at once into imbecility and death. Such, also, would be the fate of every other animal, if removed from its native haunts and influences, and placed in others which are adverse to its nature. But what is the latitude essential to man? What are the limits of his range? He has no limits. Spreading his sails on every sea, he explores every region, he pitches his tent in every clime, he braves every variety of physical circumstance. Led by the incentives of commerce, lured by the hope of gain, or seduced by the love of glory, he traverses the burning deserts of Africa, or pursues the sea-monsters in the icy regions of the pole. He hunts the tiger for pastime on the plains of India, and the buffalo for food on the prairies of the far west. He grapples with the ocean tempest, and plays with the winds of Heaven; and he even contends with the grim messenger of death amidst the fumes of plague and pestilence, and turns aside the shaft that was aimed at the bosom of his fellow man.

"Connected with this department of our subject, let us next advert briefly to the immense preponderance of the moral and intellectual faculties in man; those godlike attributes which place him on the pinnacle of creation, and convince him, as he looks down on the herd of inferior animals, that there is, indeed, a gulf between him and them. It is not for me, in this place, to dwell on these deeply interesting considerations; yet when we consider them in connexion with the gift of speech, and note the beautiful and elaborate results of human thought,—the ideality of Homer, the philosophy of Franklin, the combination of clear perception and prompt action in the immortal Washington,—when we reflect on the mighty genius that could conceive the Pyramids and the Parthenon,—or last, not least, when we contemplate that spirit of benevolence which characterizes the mind of man, and behold it directed to the mitigation of human ills, the suppression of human wrongs, and the general welfare of the human species, are we not compelled to admit that MAN, with all his faults, wtth all his manifold and glaring imperfections, possesses within him a spark of that Diviner Essence, which, if cultivated and cherished, approximates in degree to Divinity itself? Yet as this question would involve a more extended discussion than our time will admit of, I can only glance at it in very general terms. In the first place, then, we are struck with the fact, that the northern portions of both hemispheres are much more favourable to mental developement, than the corresponding southern latitudes; and that the inter-tropical regions, as Dr. Lakey has shown, may be included within the limits of this remarkable exception. In those regions of perpetual summer, wherein the mind luxuriates in indolence and sensuality, the depressing and continued heat tends to throw a languor over the human frame, alike unfavourable to mental culture and to physical activity. In vain we look, in these burning climes, for exalted manifestations of intellect, whether shown in mechanical ingenuity, in mathematical research, in philosophical inquiry, or in the more seductive themes of poesy and song. I must, of course, make exceptions for the peninsular regions of India and Arabia, which lying in the northern section of the torrid zone, present an approximation, though still remote, to the mightier genius of the people beyond the tropic.

"You may, perhaps, inquire respecting Egypt—the cradle of civilization—the mother of the arts. But it is a curious fact, I might say, coincidence, that the line of the northern tropic passes a little to the south of the island of Philœ, (which was the boundary between Egypt and Nubia,) thus placing the whole of the proper Egyptian territory within the limits of the north temperate zone. If we direct our attention yet further south to Ethiopia, we find, it is true, temples, tombs, and pyramids; but these, although more in number, are much less in magnitude than those mightier monuments which shadow the valley of the Nile from Philœ to the Delta. Again, Ethiopia was for ages a subject province or dependency of Egypt, and perhaps derived from the latter country, her arts, her language, and her religion. In the south temperate zone, notwithstanding the milder nature of the climate, and the seeming adaptation of the country, to the wants and the impulses of humanity, what do we behold? In New Holland, the lank, ferocious, and denuded savage, the feeble intelligence, the mere glimmer of the reasoning mind. In Africa, the wretched and brutalized Hottentot, yet more degraded, if possible, than the Australian himself, and constituting the lowest natural link in the scale of the rational creation. If, again, we turn our eyes to Terra del Fuego, the southern extreme of our own continent, we see another series of similar phenomena, all tending to prove that man in those regions is mentally and morally inferior to him of the inter-tropical latitudes; while both fall below the type of mind as seen in those happier climes which lie north of the tropic of cancer.

"But while much is due to climate, even more is attributable to those primeval attributes of mind, which, for wise purposes, have given our race a decided and unquestionable superiority over all the nations of the earth. Was this not the case, the numberless hordes of the Mongolians of Asia, would long since have exterminated the Caucasian race from Europe; and the very religion which we profess, would ere now have been replaced by those unhallowed rites, and that multiform idolatry, which are justly abhorrent to christianized man. Was it not for this same mental superiority, these happy climes which we now inhabit would yet be possessed by the wild and untutored Indian, and that soil which now rejoices the hearts of millions of freemen, would be yet overrun by lawless tribes of contending Barbarians. Thus it is that the white race has been able to plant and to sustain its colonies in every region of the habitable earth.

"In Asia, in Africa, in America, in the torrid and in the frigid zones, have not all the other races of men yielded and given place to this one? In the Mongolian family civilization was early, its progress was slow, and its degree is fixed. What it has been for ages, it is now. But the Caucasian stock, as seen in the Egyptians, the Chaldeans, the Greeks, the Romans, in the various nations of southern Europe, and of later time in the Anglo-Saxon race to which we ourselves belong, has marched onwards from one degree of cultivation and refinement to another, which the other races have never approached, nor are likely, in the ordinary course of events, ever to realise."

